# Microneurographic research in women

**DOI:** 10.3389/fphys.2012.00278

**Published:** 2012-07-18

**Authors:** Qi Fu

**Affiliations:** Institute for Exercise and Environmental Medicine, Texas Health Presbyterian Hospital Dallas, and UT Southwestern Medical CenterDallas, TX, USA

**Keywords:** muscle sympathetic nerve activity, arterial pressure, sympathetic neural control

## Abstract

This article reviews microneurographic research on sympathetic neural control in women under both physiological and pathophysiological conditions across the lifespan. Specifically, the effects of sex, age, race, the menstrual cycle, oral contraceptives, estrogen replacement therapy, and normal pregnancy on neural control of blood pressure in healthy women are reviewed. In addition, sympathetic neural activity during neurally mediated (pre)syncope, the Postural Orthostatic Tachycardia Syndrome (POTS), obesity, the Polycystic Ovary Syndrome (PCOS), gestational hypertension, and preeclampsia, chronic essential hypertension, heart failure, and myocardial infarction in women are also reviewed briefly. It is suggested that microneurographic studies provide valuable information regarding autonomic circulatory control in women of different ages and in most cases, excessive sympathetic activation is associated with specific medical conditions regardless of age and sex. In some situations, sympathetic inhibition or withdrawal may be the underlying mechanism. Information gained from previous and recent microneurographic studies has significant clinical implications in women's health, and in some cases could be used to guide therapy if more widely available.

## Introduction

Microneurography was developed in the mid-1960s in Sweden for percutaneous recordings of action potentials in human peripheral nerves (i.e., tibial, peroneal, median, or radial nerve), mostly in groups of sympathetic fibers (multiunit activity) but also in single axons (single-unit activity) (Wallin, 2012). In this review, we only focus on multiunit recordings. By using this technique, postganglionic efferent nerve discharges leading to the skeletal muscle (the muscular bed), which is called muscle sympathetic nerve activity (MSNA) can be recorded (Wallin et al., 1974). Criteria for adequate MSNA recordings include (1) pulse synchrony; (2) facilitation during the hypotensive phase of the Valsalva maneuver, and suppression during the hypertensive overshoot after release; (3) increases in response to breath holding; and (4) insensitivity to emotional stimuli (Wallin et al., 1974). The number of sympathetic bursts per minute (burst frequency), the number of bursts per 100 heart beats (burst incidence), and the number of bursts multiplied by mean burst area of the integrated neurogram (total activity) are used as quantitative indexes (Vallbo et al., 1979; Halliwill, 2000; Wallin, 2012). The advantages and limitations of microneurography have previously been reviewed (Wallin and Charkoudian, 2007).

Over the past 20 years or so, microneurographic studies have been performed in women of different ages under various physiological and pathophysiological conditions. Microneurography has become a potent and useful tool in clinical autonomic function assessment (Mano et al., 2006). Information gained from previous and recent microneurographic research has helped us better understand sympathetic neural mechanisms in women's health and disease.

## Physiological conditions

### Impacts of sex and age on MSNA

**Figure 1:**
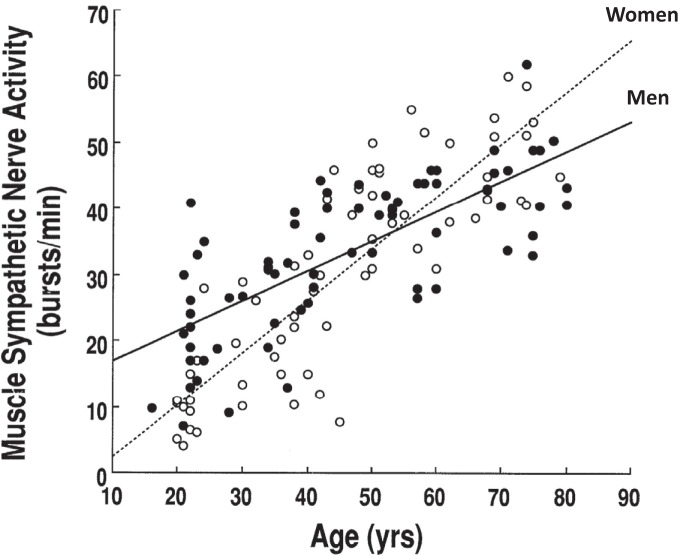
**Resting muscle sympathetic nerve activity increases with age and the increment is greater in women than in men.** Adapted with permission from Matsukawa et al. (1998b).

There are sex and age-related differences in the prevalence of hypertension and cardiovascular disease in humans. One of the important determinants for these differences is sympathetic neural control. Numerous studies have indicated that sex and/or age affect sympathetic neural activity at rest or during perturbations. For example, basal MSNA has been found to be lower in young women compared to young men (Matsukawa et al., 1998b; Narkiewicz et al., 2005). MSNA increases with age, and the increment is greater in women than in men (Iwase et al., 1991; Ebert et al., 1992; Fagius and Wallin, 1993; Matsukawa et al., 1998b; Narkiewicz et al., 2005). Therefore, elderly women have a similar or greater basal MSNA and a greater increase in arterial pressure for a given increase in MSNA than elderly men (Matsukawa et al., 1998b; Narkiewicz et al., 2005; Studinger et al., 2009; Hart et al., 2011; Okada et al., 2012b) (Figure 1). The latter may be one of the mechanisms for sex differences in hypertension in seniors (Fu et al., 2008b).

Baroreflex control of MSNA or sympathetic baroreflex sensitivity has been reported to be similar between young men and women (Tank et al., 2005; Fu et al., 2009; Studinger et al., 2009; Hart et al., 2011). With women of more advanced age, sympathetic baroreflex sensitivity remains unchanged (Ebert et al., 1992; Studinger et al., 2009) or decreases (Matsukawa et al., 1998a; Hart et al., 2011). A recent study showed that sympathetic baroreflex sensitivity was lower in elderly women than elderly men, which was associated with greater arterial stiffness in women (Okada et al., 2012b), perhaps leading to less baroreceptor distortion for a given pressure pulse.

**Figure 2:**
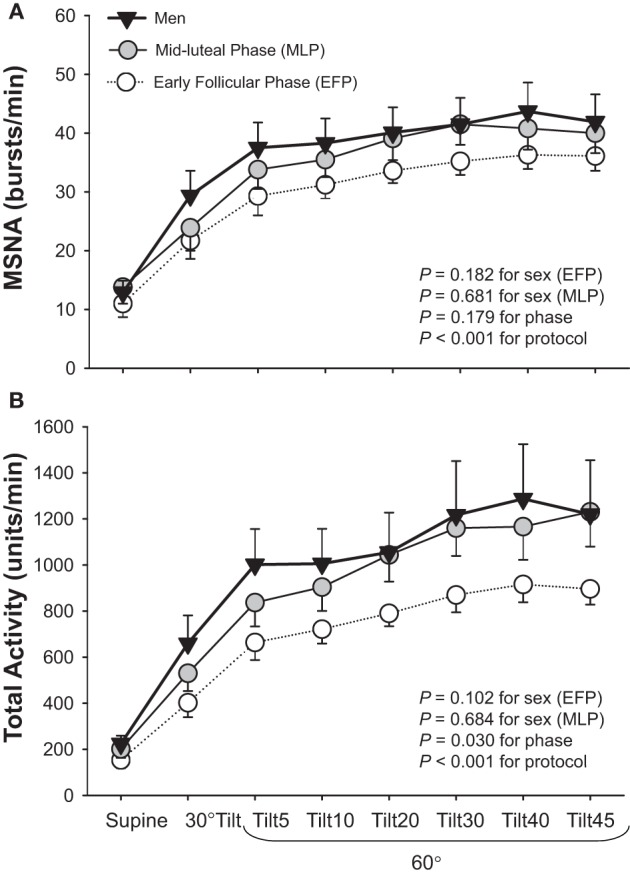
**Muscle sympathetic nerve activity (MSNA) burst frequency (A) and total activity (B) responses during a graded upright tilt in men and women during the early follicular phase (when both estrogen and progesterone are low) and the mid-luteal phase (when both sex hormones are high).** Values are means ± S.E.M. Tilt5, Tilt10, Tilt20, Tilt30, Tilt40, and Tilt45, 5, 10, 20, 30, 40, and 45 min after 60° upright tilt. Adapted with permission from Fu et al. (2009).

Studies regarding the effects of sex on sympathetic neural responses during orthostasis are few and the results are inconsistent; similar (Fu et al., 2005, 2009) or attenuated (Shoemaker et al., 2001) MSNA responses have been reported in young women compared to young men. One explanation for these inconsistent findings may be the impact of the menstrual cycle. Two of the three studies (Shoemaker et al., 2001; Fu et al., 2005) did not control for menstrual phases in female subjects. It was found that women during menstruation (i.e., the early follicular phase, low estrogen and progesterone) tended to have smaller increases in MSNA total activity (*P* = 0.102 for sex) but similar increases in burst frequency during orthostasis compared to men; however, these differences were small and not statistically robust (Fu et al., 2009) (Figure 2).

With regard to other well characterized stimuli such as static handgrip, similar (Jones et al., 1996b) or attenuated (Ettinger et al., 1996; Jarvis et al., 2011) MSNA responses have been reported in young women than young men. Jones et al. found that women and men responded with comparable increases in MSNA during 1 min of handgrip (Jones et al., 1996b). However, a common metabolic endpoint (i.e., fatigue) is necessary for comparisons between the sexes and/or age. Recent work from our laboratory showed that young women demonstrated attenuated increases in MSNA during static handgrip to fatigue and post-exercise circulatory arrest, indicating a blunted metaboreflex (Jarvis et al., 2011). Conversely, during a cold pressor test, heart rate and MSNA responses were similar in men and women, suggesting that central integration and the efferent pathway were comparable between the sexes (Jones et al., 1996b; Jarvis et al., 2011). During mental stress, young women demonstrated a smaller increase in arterial pressure than young men, but changes in MSNA and heart rate were not different between the sexes (Carter and Ray, 2009).

Taken together, sympathetic neural control can be affected by sex and/or age in humans. Young women during menstruation tend to have attenuated increases in MSNA total activity but not burst frequency during orthostasis as compared to young men, while these differences are not statistically robust. On the other hand, young women have a blunted exercise pressor reflex, which may protect them from developing hypertension and cardiovascular disease. It should be noted that the MSNA difference between men and women is much smaller than the range of MSNA observed in healthy individuals (Wallin et al., 1993; Charkoudian et al., 2005; Wallin, 2007; Joyner et al., 2010). Thus, the influences of genes, age, or other factors may be much greater when compared to a sex effect. The greater age-related increase in basal MSNA and decrease in baroreflex function may be responsible for the high prevalence of hypertension and cardiovascular disease in elderly women.

### Racial differences in MSNA

Black women have a higher prevalence of hypertension compared to white women (Burt et al., 1995), which may be due to racial differences in sympathetic neural control of blood pressure. It was previously found that physiological or psychological stressors increased MSNA more in young blacks than in young whites (Calhoun, 1992; Calhoun et al., 1993), especially in those with a family history of hypertension (Calhoun and Mutinga, 1997). Conversely, some investigators showed that there were no significant differences in basal or stimulated norepinephrine spillover between young blacks and whites (Stein et al., 2000). Ray et al. found that compared to young whites, young blacks had lower MSNA responses but similar vasoconstriction during orthostatic stress, indicating an enhanced sympathetic vascular transduction in blacks (Ray and Monahan, 2002). Consistent with the study of Ray et al., we recently found that elderly blacks had a blunted sympathetic neural responsiveness but greater pressor response during orthostasis compared with elderly whites (Okada et al., 2012a). However, sex was not investigated in this study.

Although at rest young lean black men were found to have a greater MSNA than white men, basal MSNA was similar between young black and white women (Abate et al., 2001). In black women, adiposity seems to be a major determinant of sympathetic discharge (Abate et al., 2001). It is possible that black women have enhanced sympathetic neural responses and/or sympathetic adrenergically medicated vasoconstriction during perturbations compared to white women, which may be the mechanisms for the higher prevalence of hypertension in this population.

Racial differences in sympathetic neural control in women have not been investigated extensively. There is no clear synthesis possible at this time.

### Menstrual cycle effects and oral contraceptives in premenopausal women

Results regarding the effects of the menstrual cycle on sympathetic neural control in healthy young women are controversial. Some (Minson et al., 2000a; Park and Middlekauff, 2009) but not all (Jones et al., 1996b; Carter et al., 2009; Fu et al., 2009; Jarvis et al., 2011) investigators have reported that the fluctuations of female sex hormones during the menstrual cycle may affect basal MSNA and sympathetic baroreflex sensitivity at supine rest in women. Two studies have uniformly demonstrated that the menstrual cycle can affect MSNA responses during orthostasis (Carter et al., 2009; Fu et al., 2009). For example, it was found that an increase in MSNA total activity during upright tilt (Fu et al., 2009) and lower body negative pressure (Carter et al., 2009) was smaller in the early follicular phase (day 1–5 after the onset of menstruation, low estrogen and progesterone) than in the mid-luteal phase (day 19–22, high estrogen and progesterone). However, both studies showed that the menstrual cycle did not influence sympathetic baroreflex sensitivity responses during orthostatic stress (Carter et al., 2009; Fu et al., 2009). On the other hand, MSNA responses during static handgrip (Minson et al., 2000a; Jarvis et al., 2011), mental stress (Carter and Lawrence, 2007), and vestibulosympathetic stimulation (Lawrence et al., 2008) were all similar between menstrual phases.

To date, there are only two studies published regarding the effects of oral contraceptives on sympathetic neural control in young women. Minson et al. previously showed that oral contraceptive use did not affect basal MSNA, but it affected resting sympathetic baroreflex sensitivity (Minson et al., 2000b). Furthermore, the authors found that changes in baroreflex sensitivity with oral contraceptive use differed from changes in baroreflex sensitivity during the normal menstrual cycle (Minson et al., 2000b). Conversely, Carter et al. showed that oral contraceptives did not alter cardiovascular and sympathetic neural responses to an orthostatic challenge in young healthy women (Carter et al., 2010). Whether similar observations can be made during other perturbations such as the cold pressor test and mental stress needs to be determined.

Taken together, recent studies have shown that the menstrual cycle can affect MSNA total activity responses during orthostatic stress, and women during the early follicular phase have attenuated upright total activity. However, sympathetic neural responses during static handgrip, mental stress, and vestibulosympathetic stimulation are not influenced by the menstrual cycle. Oral contraceptives appear to have limited impact on sympathetic neural control in young women. Further research is needed to confirm these findings.

### Impacts of estrogen replacement therapy on MSNA in postmenopausal women

Menopausal estrogen loss is associated with an increased risk for cardiovascular events in women (Burt et al., 1995). Supplemental estrogen has been proposed to reverse, to some extent, deficits in blood pressure control and protect against the development of hypertension and cardiovascular disease in postmenopausal women (Hunt et al., 2001). However, the therapeutic effects of estrogen supplementation may be influenced by the dosage, the type of estrogen used, the duration of treatment (i.e., acute versus chronic) and/or the route of administration (i.e., transdermal versus oral). For example, low-dose estrogen replacement therapy was found to be not effective in decreasing MSNA and blood pressure in postmenopausal women (Oneda et al., 2008). Transdermal administration of estrogen supplement suppressed basal MSNA without augmenting arterial baroreflexes, and sympathetic inhibition was evident only with chronic rather than acute estrogen administration (Vongpatanasin et al., 2001; Weitz et al., 2001). Conversely, oral administration of estrogen does not change basal levels of MSNA (Hunt et al., 2001; Vongpatanasin et al., 2001; Moreau et al., 2003).

These studies suggest that chronic transdermal administration of estrogen supplement at a high dosage may be necessary in the restoration of the deficits in sympathetic neural control of blood pressure in postmenopausal women.

### Sympathetic neural control during normal pregnancy

Normal pregnancy is associated with dramatic changes in hemodynamics, which occur through autonomic control mechanisms (Ekholm et al., 1994; Fu and Levine, 2009). Earlier human studies on sympathetic activity during pregnancy focused only on plasma norepinephrine concentrations, which ranged from increased to decreased compared with non-pregnant conditions (Zuspan, 1979; Tunbridge and Donnai, 1981; Barron et al., 1986; Chapman et al., 1998). With microneurography, Greenwood et al. found that basal MSNA increased in normotensive pregnant women during the third trimester of gestation (Greenwood et al., 1998, 2001). A decrease in baroreceptor-mediated inhibitory restraint on central sympathetic outflow may be one potential explanation for these findings. It is also possible that changes in hormonal factors associated with pregnancy may cause sympathetic activation. Greenwood et al. followed three normotensive pregnant women 6 weeks after delivery and found that sympathetic activity decreased dramatically (Greenwood et al., 1998). Their preliminary data indicate that the end of normal pregnancy may be associated with an increase in resting sympathetic outflow and pregnancy *per se* may result in sympathetic activation despite a normal blood pressure.

We recently found that basal MSNA and MSNA in the upright posture were markedly greater, while diastolic pressure was lower and peripheral vascular resistance decreased during early pregnancy (i.e., ≤ 8 weeks of gestation) compared to pre-pregnancy in healthy young women (Jarvis et al., 2012). These results suggest that sympathetic activation may be a common characteristic of *early* pregnancy in humans. These observations challenge conventional thinking about blood pressure regulation during pregnancy, which suggests that sympathetic activation occurs only at the end of pregnancy, and to our knowledge, there are no published sympathetic nerve recordings during *early* pregnancy in humans. Our study has shown that marked sympathetic activation occurs within the first few weeks of conception, and may provide the substrate for pregnancy induced cardiovascular complications. If we confirm that women with hypertensive disorders of pregnancy have a greater increase in MSNA or sympathetic vasoconstriction *early on* during pregnancy when compared to women with normotensive pregnancies, we could make an early diagnosis or prediction. Early prevention or treatment targeted to the appropriate pathophysiology may be initiated, which may reduce maternal and fetal death or morbidity, as well as cardiovascular risks in women later in life.

Taken together, sympathetic activation is a common phenomenon during normal pregnancy in healthy women, which occurs as early as 4 weeks of gestation, remaining high throughout the entire pregnancy. It may represent a compensatory mechanism induced by peripheral vasodilation; in addition, alterations in hormonal factors associated with pregnancy may also contribute to sympathetic activation in pregnant women.

## Pathophysiological conditions

### Syncope and sympathetic withdrawal

Syncope is a common clinical condition affecting up to 40% of otherwise healthy people (Ganzeboom et al., 2006; Serletis et al., 2006), especially young women (Robertson, 1999; Fu et al., 2004). Based on current conventional wisdom, loss of sympathetic tone (Wallin and Sundlof, 1982; Dietz et al., 1997; Morillo et al., 1997; Mosqueda-Garcia et al., 1997) with relaxation of vascular resistance (Lewis, 1932; Barcroft et al., 1944; Weissler et al., 1957; Epstein et al., 1968) is thought to play a major role in neurally mediated syncope. However, Cooke et al. reported that withdrawal of MSNA was not a prerequisite for (pre)syncope despite significant decreases of arterial pressure in healthy individuals (Cooke et al., 2009). We recently showed that MSNA decreased rapidly at presyncope often after the onset of hypotension, while a moderate fall in cardiac output with coincident vasodilation or a marked fall in cardiac output with no changes in peripheral vascular resistance may contribute to (pre)syncope (Fu et al., 2012). Blood pressure and MSNA responses in the development of presyncope seem to be similar between young men and young women, as well as between menstrual phases in women (Fu et al., 2009).

In a retrospective study in healthy young individuals without manifest cardiovascular disease, we found that approximately 10–15% of the presyncopal subjects (all females) had no significant decreases in MSNA prior to presyncope, while these subjects appeared to have greater increases in plasma epinephrine concentration (Fu et al., 2008a), suggesting that stimulation of β_2_-adrenergic receptors without changes in vasomotor sympathetic activity may also elicit (pre)syncope in humans, though this must be tested in a prospective design.

Taken together, sympathetic withdrawal at (pre)syncope occurs in the majority of healthy people without manifest cardiovascular disease. In a small subset, stimulation of β_2_-adrenergic receptors without sympathetic withdrawal may elicit (pre)syncope. Recent findings suggest that sympathetic withdrawal may not be a prerequisite for (pre)syncope, because MSNA decreases rapidly at presyncope after the onset of hypotension. A moderate fall in cardiac output with coincident vasodilation or a marked fall in cardiac output with no changes in peripheral vascular resistance may contribute to (pre)syncope in this population. These patterns may uncover distinct patient populations with different mechanisms and could be a target for pathophysiology specific therapy if borne out in clinical trials.

### Neural control in the postural orthostatic tachycardia syndrome

The Postural Orthostatic Tachycardia Syndrome (POTS, also called Chronic Orthostatic Intolerance) affects >500, 000 Americans, the vast majority of whom are premenopausal women (Robertson, 1999). POTS is characterized by orthostatic tachycardia without significant hypotension (Raj et al., 2005). Patients with POTS were previously reported to have altered baroreflex function (Stewart, 2000), hyperadrenergic activity (Jacob et al., 1997), or postganglionic sympathetic denervation and inadequate peripheral vasoconstriction (Stewart and Weldon, 2000).

Microneurographic studies have shown that POTS patients have a similar (Bonyhay and Freeman, 2004; Muenter Swift et al., 2005; Lambert et al., 2008; Fu et al., 2010) or greater (Furlan et al., 1998) basal MSNA when compared to healthy controls. All (Muenter Swift et al., 2005; Lambert et al., 2008; Fu et al., 2010; Baumert et al., 2011) except one (Furlan et al., 1998) studies reported enhanced MSNA responses during orthostasis in POTS. Sympathetic baroreflex sensitivity at rest was similar (Bonyhay and Freeman, 2004; Fu et al., 2010) or even greater (Bonyhay and Freeman, 2004; Muenter Swift et al., 2005) in POTS patients than healthy controls. MSNA responses during static handgrip and the cold pressor tests were comparable between patients and controls, even though absolute values of heart rate were consistently higher in POTS (Fu et al., 2010).

Taken together, these results indicate that modulation of the sympathetic nervous system by the baroreflex, muscle metaboreflex, mechanoreflex, and central command may be intact in patients with POTS.

### Sympathetic neural activity in obesity

Obesity is associated with an increased risk of hypertension and cardiovascular disease in humans. It has been proposed that the sympathetic nervous system plays a role in obesity, while ethnicity may alter the relationship between sympathetic activity and obesity (Spraul et al., 1993; Weyer et al., 2000). For example, it was found that in Pima Indians there was no relationship between an increase in sympathetic activity and an increase in adiposity, which may contribute to the low prevalence of hypertension in this population (Weyer et al., 2000).

Jones et al. found that percent body fat was related to basal MSNA in both young men and women, but the regression line was shifted downward in women because of lower levels of MSNA (Jones et al., 1996a). They also reported that waist-to-thigh ratio was a better correlate of MSNA than percent body fat; MSNA and waist-to-thigh ratio were correlated significantly in men but not as strongly in women. These observations were confirmed by the study of Tank et al. showing that adjusting for age, waist circumference, waist-to-hip ratio, and body mass index were predictive for MSNA in men but not in women (Tank et al., 2008). These results suggest that abdominal fat is an important adipose tissue depot regulating MSNA in men, while women may be protected from sympathetic activation through a hitherto unknown mechanism (Tank et al., 2008).

Nevertheless, obesity was found to be a major determinant of sympathetic discharge in women (Abate et al., 2001). Obese normotensive women had a greater basal MSNA than lean women (Negrao et al., 2001; Ribeiro et al., 2001; Lambert et al., 2007). However, they had smaller increases in MSNA during static handgrip and post-exercise circulatory arrest compared with age-matched lean women, indicating a blunted metaboreflex in obesity (Negrao et al., 2001). Body weight reduction in obese women resulted in a decrease of blood pressure that was at least partially caused by a reduction of MSNA (Andersson et al., 1991). It was also found that weight loss by diet or diet plus exercise training reduced MSNA during mental stress in obese women (Tonacio et al., 2006). An improvement in baroreflex control, decreases in free fatty acids and plasma leptin levels, or an increase in insulin sensitivity after weight loss may be responsible for the reduction of MSNA (Tonacio et al., 2006).

Taken together, the sympathetic nervous system plays a role in obesity in both men and women, but ethnicity may alter the relationship between sympathetic activity and obesity. Waist-to-hip ratio correlates with MSNA in men but not in women. Nevertheless, obese women have a greater level of basal MSNA compared to lean women. In obese women, weight loss decreases blood pressure, at least in part, due to a reduction in MSNA.

### Polycystic ovary syndrome

Polycystic ovary syndrome (PCOS) is the most common endocrine abnormality in women of reproductive age characterized by androgen excess and hyperinsulinemia (Schlaich et al., 2011). It has been proposed that PCOS is a state of sympathetic overactivity (Lara et al., 1993; Sverrisdottir et al., 2008). Testosterone was found to be a predictor of sympathetic nerve activity in women with PCOS and increased basal MSNA was associated with a higher level of testosterone in these patients (Sverrisdottir et al., 2008). A recent case report in two obese PCOS patients with hypertension showed that renal denervation exerted beneficial effects not only on blood pressure control but also on insulin resistance, renal, and endocrine abnormalities characteristic of PCOS (Schlaich et al., 2011). Three months after the denervation, basal MSNA decreased in both patients (Schlaich et al., 2011). Additionally, low-frequency electroacupuncture, and physical exercise could reduce MSNA and improve clinical symptoms in women with PCOS (Stener-Victorin et al., 2009).

Thus, patients with PCOS have sympathetic overactivity, which may be attributable to increased levels of testosterone. Interventions that can reduce sympathetic activity may improve symptoms in these patients.

### Gestational hypertension and preeclampsia

**Figure 3:**
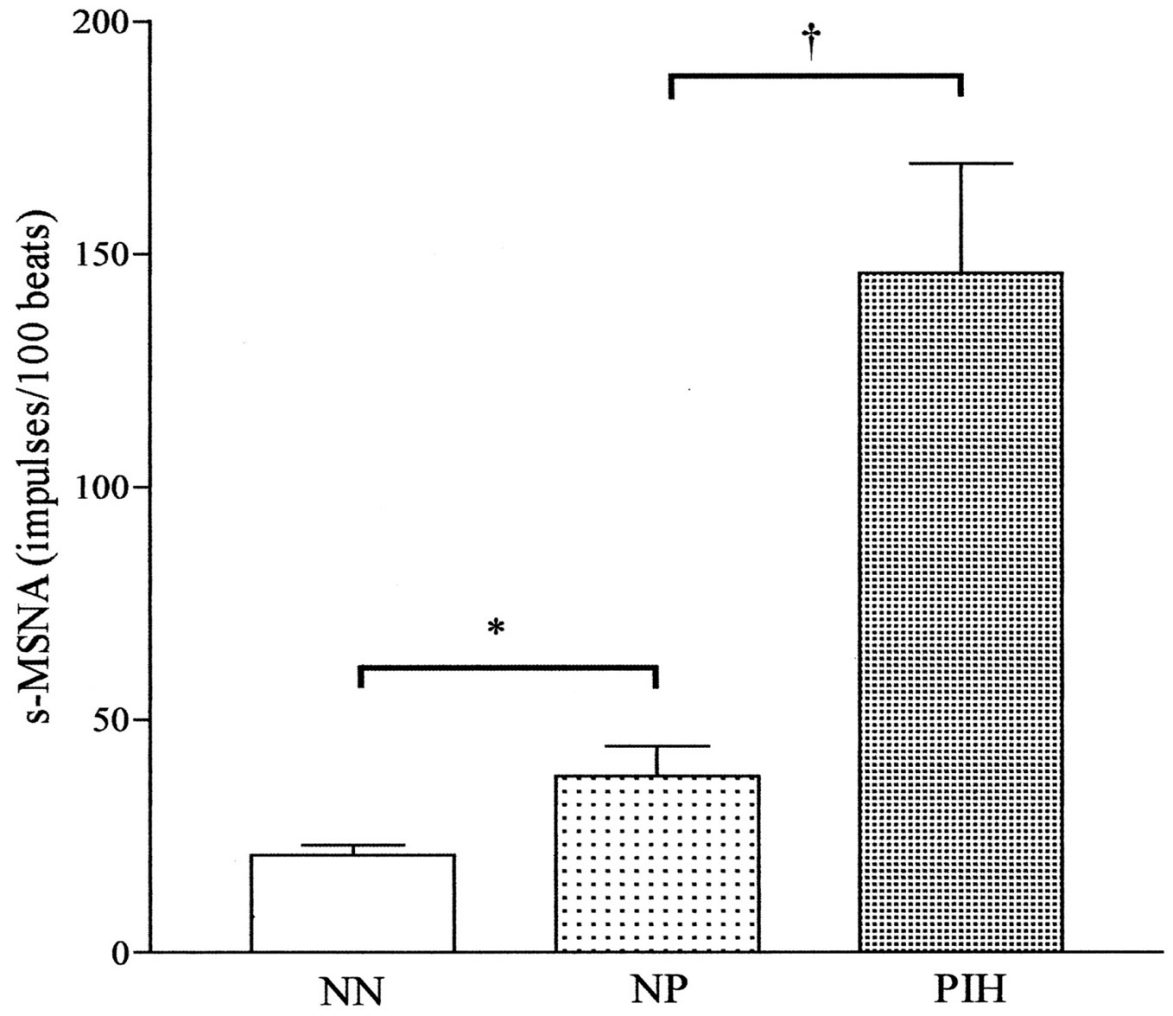
**Resting mean frequency of single-unit muscle sympathetic nerve activity (s-MSNA) in the three groups of subjects, NN, NP, and PIH, expressed as mean (height of columns) and S.E.M. (bars).** NN, normotensive non-pregnancy; NP, normotensive pregnancy; PIH, pregnancy-induced hypertension. ^*^P < 0.05; ^†^*P* < 0.001. Adapted with permission from Greenwood et al. (2001).

Central sympathetic outflow has been found to increase in women with normal pregnancies and is even greater in hypertensive pregnant women during *late* pregnancy (Greenwood et al., 1998, 2001, 2003) (Figure 3). These findings suggest that sympathetic activation during the latter months of pregnancy may help to return arterial pressure to non-pregnant levels, and when the increase in activity is excessive, hypertension may occur. This notion is supported by the findings of Schobel et al. showing that preeclamptic women had excessive sympathetic overactivity (i.e., more than three times as high as that in normotensive pregnant women) during late pregnancies (Schobel et al., 1996). It has been proposed that pregnancy induced sympathetic overactivity constitutes a precursor of preeclampsia, which is physiologically compensated for by vasodilating mechanisms, leading to preeclampsia only when they fail (Fischer et al., 2004). However, it remains unclear whether sympathetic overactivity is a cause or consequence of hypertensive disorders in pregnancy.

A previous study showed that preeclampsia was not associated with greater sympathetic hyperactivity than gestational hypertension, suggesting that any renal impairment in preeclampsia involves mechanisms that are not solely dependent on sympathetic hyperactivity (Greenwood et al., 2003). In patients with preeclampsia, epidural anesthesia reduces uterine artery vascular resistance (Ramos-Santos et al., 1991; Ginosar et al., 2009). These results indicate that sympathetic vasoconstriction may be specifically augmented in preeclamptic women. However, it is completely unknown whether women with gestational hypertension and preeclampsia have excessive sympathetic activation and/or enhanced sympathetic vasoconstriction early on during pregnancy, or whether sympathetic overactivity occurs only at term, providing the substrate for both of these complications.

Taken together, both gestational hypertension and preeclampsia are associated with sympathetic overactivity. However, whether sympathetic overactivity is a cause or consequence of hypertensive disorders during pregnancy remains to be determined.

### Essential hypertension, heart failure and myocardial infarction

Women with chronic essential hypertension have increased levels of basal MSNA compared with their normotensive counterparts, and MSNA is significantly related to blood pressure (Lambert et al., 2007). However, young and middle-aged (mean age 51 years old) female hypertensive patients have a lower level of sympathetic activity compared to aged-matched male patients (Hogarth et al., 2007). Postmenopausal women (mean age 58 years old) continue to have a lower level of basal MSNA than similarly aged men even after the development of chronic essential hypertension (Hogarth et al., 2008). Whether older (i.e., ≥ 65 years old) and/or very old (i.e., ≥ 80 years old) hypertensive women have a similar or even greater MSNA compared with age-matched hypertensive men needs to be investigated.

Recent studies have shown that basal MSNA is not clearly different between middle-aged female and male patients with chronic heart failure (Antunes-Correa et al., 2010). The benefits of exercise training on neurovascular control and functional capacity in patients with heart failure are independent of sex (Antunes-Correa et al., 2010).

Sympathetic activation occurs following acute myocardial infarction (Karlsberg et al., 1981; McAlpine et al., 1988; Graham et al., 2002; Hogarth et al., 2009) and is related to the extent of myocardial damage (McAlpine et al., 1988; Hogarth et al., 2009). Following uncomplicated acute myocardial infarction, women were found to develop a relatively greater magnitude of sympathetic activation lasting until its resolution at 9 months. This is consistent with reports of their worse prognosis observed during this time period with important potential clinical implications (Hogarth et al., 2009).

Taken together, sympathetic activation is associated with hypertension and cardiovascular disease in both women and men. Sex may affect the degree of sympathetic activation and the prognosis of the disease. Such information may be useful for selection of treatment for female patients.

## Summary

Previous and recent microneurographic studies suggest that sympathetic neural control may be affected by physiological changes such as age, race, fluctuations of female sex hormones during the menstrual cycle, use of oral contraceptives, and pregnancy. In additional, pathophysiological conditions such as syncope, POTS, obesity, PCOS, hypertensive pregnancy, essential hypertension, heart failure, and myocardial infarction may also affect sympathetic neural control in women. In many medical conditions, excessive sympathetic activation is a primary or secondary underlying cause regardless of sex and age. In some situations, for example, syncope, sympathetic inhibition or withdrawal may be one of the determinant factors. Results obtained from these studies provide valuable information regarding women's health and disease, which has significant clinical implications and is relevant to public health.

### Conflict of interest statement

The author declares that the research was conducted in the absence of any commercial or financial relationships that could be constructed as a potential conflict of interest.
